# Construct validity of the Mini-BESTest in individuals with chronic pain in specialized pain care

**DOI:** 10.1186/s12891-023-06504-9

**Published:** 2023-05-17

**Authors:** Sofia Wagner, Annika Bring, Pernilla Åsenlöf

**Affiliations:** Department of Women’s and Children’s Health, Uppsala University, Uppsala, Sweden

**Keywords:** Chronic pain, Specialized pain care, Balance, Mini-BESTest, Validity, Internal consistency

## Abstract

**Background:**

Balance assessment scales are important clinical tests to identify balance impairments. Chronic pain (> 3 months) is associated with impaired dynamic balance; however, very few balance assessment scales are psychometrically evaluated for the population. The purpose of this study was to evaluate the construct validity and internal consistency of the Mini-BESTest for individuals with chronic pain in specialized pain care.

**Methods:**

In this cross-sectional study, 180 individuals with chronic pain (> 3 months) were assessed with the Mini-BESTest and included in the analyses. For construct validity, five alternative factor structures were evaluated using a confirmatory factor analysis. In addition, we tested the *a priori* hypotheses about convergent validity with the 10-meter walk test, and divergent validity with the Brief Pain Inventory (BPI): pain intensity, the Tampa Scale of Kinesiophobia-11 (TSK-11), and the Pain Catastrophizing Scale (PCS-SW). Internal consistency was evaluated for the model with the best fit.

**Results:**

A one-factor model with added covariance via the modification indices showed adequate fit indices. In line with our hypotheses, Mini-BESTest showed convergent validity (r_s_ = > 0.70) with the 10-meter walk test, and divergent validity (r_s_ = < 0.50) with BPI pain intensity, TSK-11, and PCS-SW. Internal consistency for the one-factor model was good (α = 0.92).

**Conclusions:**

Our study supported the construct validity and internal consistency of the Mini-BESTest for measuring balance in individuals with chronic pain, who were referred to specialized pain care. The one-factor model showed an adequate fit. In comparison, models with subscales did not reach convergence, or showed high correlations between subscales, implying that Mini-BESTest is measuring one construct in this sample. We, therefore, propose using the total score, instead of subscale scores, for individuals with chronic pain. However, further studies are necessary to establish the reliability of the Mini-BESTest in the population.

**Supplementary Information:**

The online version contains supplementary material available at 10.1186/s12891-023-06504-9.

## Introduction

Individuals with chronic pain, i.e., primary or secondary pain > 3 months [1], describe having balance problems [2] and falls in their everyday lives [3, 4]. Several studies indicate that chronic pain is associated with impaired dynamic balance [5, 6], even so, how balance affects daily functioning and how to identify individuals with balance impairments in pain care are less studied areas.

Balance assessment scales, based on a set of functional tests, are used more and more in clinical pain research. The Mini Balance Evaluation Systems Test (Mini-BESTest) [6–8], the Balance Evaluation Systems Test (BESTest) [3], and the Berg balance scale [9] are examples of scales that have been used to explore balance, functional performance, or as an outcome measure in individuals with chronic pain. The Mini-BESTest includes 14 items measuring dynamic balance, divided into four subscales [10]. The scale was originally developed for adults with diverse neurological diagnoses and is now widely used in clinical practice and research [11]. In comparison, the Mini-BESTest requires less time than the BESTest, and it is often preferred to the Berg balance scale due to the lack of ceiling effects [11]. However, the psychometric properties of Mini-BESTest in individuals with chronic pain have not been evaluated.

When evaluating the Mini-BESTest psychometrically, it is important to consider whether the subscales reflect different constructs of balance control, i.e., if the scale is multi- or uni-dimensional. The Mini-BESTest was originally developed as a shorter version of the multi-dimensional scale BESTest [12], and scores from the subscales are sometimes reported separately [6, 13]. Nonetheless, consistent with a conceptual framework based on the hypothesis of integrated control of posture and gait [14, 15], all items on the Mini-BESTest were originally proposed to reflect a uni-dimensional construct of dynamic balance [10]. This original model was later found to be well-fitting for data from individuals with different neurological conditions [16–19]. However, cases of a multi-dimensional model have also been suggested [20, 21].

A range of biopsychosocial factors are related to pain disability in individuals with chronic pain [22–24], and it has been suggested that factors such as pain intensity [25], fear of movement, and pain catastrophizing [26, 27] affect balance. Hence, when interpreting the results from a balance assessment, a highly important aspect is whether the balance scale can differentiate balance from other possibly related constructs, such as fear of movement, pain catastrophizing and pain intensity. Another aspect to explore to capture possible sex differences concerning balance [28] is whether the scale measures similarly for both sexes.

Thus, evaluating the Mini-BESTest psychometrically in individuals with severe pain problems is of great value for clinical care and research. Given the lack of any gold standard scales for balance assessment for individuals with chronic pain, an adequate first step is to evaluate the construct validity of the Mini-BESTest in this population [29]. Thus, this study aimed to evaluate the construct validity and internal consistency of the Mini-BESTest, Swedish version 2.1.1, for individuals with chronic pain in specialized pain care. This was done by examining its structural validity, and testing priori hypotheses to evaluate convergent and divergent validity. Another aim was to study if there were any sex differences concerning the criteria for convergent or divergent validity.

The hypotheses were formulated based on the literature and in relation to suggested cut-offs for convergent and divergent validity [30]. Consistent with the suggested conceptual model for Mini-BESTest [14, 15], we expected a convergent validity (r_s_ = ≥ 0.70, positive direction) between Mini-BESTest and walking speed (hypothesis 1). Furthermore, we hypothesized that the constructs of pain intensity, fear of movement/(re)injury, and pain catastrophizing would be related to balance, while still being different constructs [25–27]. We, therefore, expected a divergent validity (r_s_ = < 0.50, negative direction) between Mini-BESTest and Brief Pain Inventory pain intensity (hypothesis 2), followed by a weaker relationship between Mini-BESTest and the Tampa Scale of Kinesiophobia-11 (hypothesis 3) and the Pain Catastrophizing Scale (hypothesis 4), respectively.

## Methods

### Sample and setting

The study is part of the U-PAIN cohort study aiming to explore the benefits and risks of opioid use in chronic pain, more extensively described elsewhere [31]. The study was approved by the Swedish Ethical Review Authority’s regional ethics board in Uppsala (EPN Uppsala D-No 2016 − 376, 2020–05283), and complies with the Declaration of Helsinki. For the planning and reporting of the study, the COnsensus-based Standards for the selection of health Measurement Instruments (COSMIN) checklist was followed [32].

Participants for the cohort study were recruited among individuals referred to secondary or tertiary care at the Pain Center at Uppsala University Hospital. Inclusion criteria were: ≥18 years old and pain duration of ≥ 3 months at the time of the referral. Individuals receiving acute care related to active cancer treatment or palliative care, and individuals who had cognitive impairment, or were illiterate in the Swedish language, were excluded. The sample in this study includes all participants assessed using the Mini-BESTest among the first 200 participants recruited to the cohort between June 2018 and January 2021, which is considered as a sufficient sample size in relation to the planned analyses [32].

Data were collected in conjunction with the individuals’ first visit of their current referral to the pain center. All individuals received written and oral information before giving written consent to participate. Patient-reported outcome measures (PROMs) were filled in by using a personal secure log-in to the Swedish Healthcare Guide’s digital platform, within six weeks before or after the research visit. Paper and pencil versions were offered upon request. During the research visit, data on the Mini-BESTest and 10-meter walk test were collected by raters trained in the test administration.

### Measures

Data for sample characterization were collected by using a study-specific questionnaire. Pain classification, according to the International Association for the study of Pain (IASP) classification of chronic pain for the International Classification of Diseases (ICD-11) [1], was based on retrospective data from medical records.

Mini-BESTest is proposed to measure dynamic balance using 14 items, divided into four subscales: anticipatory postural adjustments, reactive postural control, sensory orientation, and dynamic gait [10, 33]. Each item is scored on a 3-grade ordinal scale, where 0 = *unable or requiring help to perform* and 2 = *normal function.* Total scores range from 0 to 28 [10]. Two items are assessed on both the right and left side, only calculating the worse score in the total score [13]. In this study, if a participant declined to perform one or more items, these were scored with 0. Mini-BESTest has excellent test-retest reliability and inter-rater reliability in individuals with different, mainly neurological, conditions [11], as well as high concurrent validity with other balance measures in individuals with stroke and Parkinson’s disease [33]. To estimate the minimal clinically important difference for different conditions, for example, Type 2 diabetes patients with peripheral neuropathy, and patients with different neurological conditions, a change between 3 and 5 points has been suggested [34–37]. For the Swedish version, good test-retest reliability and inter-rater reliability are found in a clinical setting for individuals having Parkinson’s disease [38]. Psychometric studies on individuals with chronic pain are lacking.

For hypotheses testing, variables were selected based on previous literature on association with balance [14, 15, 25–27]. The choice of specific instruments was dependent on measures included in the comprehensive cohort study in which this study is embedded. They all represent core domains in specialized pain care.

The 10-meter walk test (10MWT) was used to measure comfortable walking speed (CWS) and maximum walking speed (MWS). For the calculation of walking speed in meters per second (m/s), an average of three trials was used [39]. An acceleration phase was applied, and a handheld stop-watch was used for timing [39]. Initial support for the test-retest reliability and interrater reliability is provided for pregnant women with pelvic girdle pain [40], as well as for using hand held stop-watches [41]. To estimate the minimal clinically important difference for older adults and individuals with neurological diseases, a change between 0.10 and 0.16 m/s has been suggested [42, 43]. Psychometric studies on individuals with chronic pain are lacking.

The Swedish version of Brief Pain Inventory - Short form (BPI-SF) was used to measure pain intensity and pain interference [44]. In the subscale pain intensity, the *current pain* and pain intensity at its *worst*, *least*, and *on average* are scored on a numeric rating scale (NRS), with anchors ranging from 0, *no pain*, to 10, *pain as bad as you can imagine*. In the subscale pain interference, the impact of pain on functioning is scored on NRSs, with anchors ranging from 0, *does not interfere*, to 10, *complete interference*. A composite mean score was computed for each subscale [45]. The interference subscale was used for the background characteristics only. The scale has been found to be reliable in pain-related musculoskeletal conditions [46]. The validity is supported in individuals in specialized pain care with chronic non-cancer pain [47]; moreover, a two-factor model with the constructs pain intensity and pain interference has been suggested [46, 48]. To estimate the minimal clinically important difference for pain measures for individuals with fibromyalgia, a change of approximately 2.2 points in the severity score has been suggested [49]. Psychometric studies for the Swedish version of the BPI-SF are lacking. In our sample, internal consistency for pain intensity was α = 0.86, which is consistent with previous studies (α = 0.85) [47].

The Tampa Scale of Kinesiophobia-11 (TSK-11) was used to measure fear of movement/(re)injury. Eleven items are individually scored on a 4-point Likert scale, where 1 = *strongly disagree* and 4 = *strongly agree* [50]. The total score ranges from 11 to 44 points [50]. Both validity and reliability are supported for individuals with chronic pain [50]. For the structural validity, a model with the two latent constructs, somatic focus and activity avoidance, is suggested to contribute to an overall second-order construct of fear of movement/(re)injury [51, 52]. For the Swedish version, the construct validity in individuals with musculoskeletal pain [51] and older individuals with chronic pain [52] is supported. The construct validity and reliability in the latter population were acceptable [52]. To identify an important reduction in the fear of movement for individuals with chronic low back pain, a change with approximately 4 points has been suggested [50]. Internal consistency for the total scale was α = 0.84 in our sample, which is consistent with previous studies, where α ranged between 0.79 and 0.87 [50, 52].

The Pain Catastrophizing Scale – Swedish version (PCS-SW) was used to measure pain catastrophizing. Thirteen items are individually scored on a 5-point Likert scale, where 0 = *never* and 4 = *all the time* [53]. The total score ranges from 0 to 52 points [53]. PCS is well-established and considered reliable [53, 54] and valid [53]. For the structural validity, a model with the three latent constructs, rumination, magnification, and helplessness, is suggested to contribute to an overall second-order construct of pain catastrophizing [53, 55]. Initial support for structural validity and internal consistency in individuals with chronic musculoskeletal pain is provided for a Swedish version [55]. For the minimal clinically important difference for individuals with chronic low back pain undergoing multidisciplinary rehabilitation, a change between 8 and 14 points has been suggested [56]. Internal consistency for the total scale was α = 0.93 in our sample, which is congruent with previous studies (α = 0.92)[54, 55].

### Statistical analyses

A confirmatory factor analysis was performed using R version 4.1.1. All other statistical analyses were performed using the IBM SPSS Statistics, version 26 (IBM Corp., Armonk, New York). All statistical tests were 2-tailed, and a *p* value of ≤ 0.05 was considered significant.

For interpretability, the number (%) of participants scored with each score (0–2), as well as the mean and SD, were calculated for all fourteen Mini-BESTest items to illustrate the distribution of the sample over the scores [29]. In addition, the number (%) of participants that declined to perform each item was calculated. To compare the respective score for each item from the group that did not perform all items with those from the group with a complete Mini-BESTest, Fisher’s exact test was used. An independent t-test was used to compare the walking speed between the two groups. The floor and ceiling effects were measured using the proportion of participants who scored with the minimum and maximum score, respectively. The floor effects were considered present if > 15% of the participants scored 0 points on the Mini-BESTest [57]. Similarly, the ceiling effects were considered present if > 15% of the participants scored 28 points [57].

A confirmatory factor analysis was conducted to evaluate the structural validity of Mini-BESTest. For the confirmatory factor analysis, diagonally weighted least squares with polychoric correlation matrix were used for modeling the Mini-BESTest items as ordinal data [58]. Following the methodology used by Godi et al. [18], five alternative models [10, 20, 21] were compared. Model 1 was based on the original one-factor model [10], where all 14 items were assumed to be related to one latent factor reflecting dynamic balance. Model 2 was a first-order 4-factor model, where the 14 items were divided into four latent factors (anticipatory postural adjustments, reactive postural control, sensory orientation, and dynamic gait) based on the four subscales. Model 3 was a second-order model, where the four first-order latent variables of model 2 were assumed to be related to the second-order latent construct dynamic balance. Model 4 was a second-order model, consisting of 13 measured items (excluding item 7) [21]. Three first-order latent factors (anticipatory postural adjustments, reactive postural control, and sensory orientation) were assumed to be related to the second-order latent construct dynamic balance. Model 5 was a first-order model similar to model 2, although consisting of 13 items (excluding item 7), divided into four latent factors (anticipatory postural adjustments, postural response, sensory orientation, and stability in gait) [20].

As a first step to consider a model as reasonably correct, we investigated if all factor loadings were at least 0.5 and ideally ≥ 0.70, the latter as an indication of the factor explaining at least half of the variance in the indicator [59]. Further, we investigated if the correlations between the factors were < 0.90 as an indication of them measuring different constructs [59, 60]. Secondly, to assess the fit of the model to the data, five indexes were used: Chi-square test (χ ^2^; acceptable fit if not significant), the root mean square error of approximation (RMSEA; < 0.05 good model fit, ≤ 0.08 adequate fit), the comparative fit index (CFI), and the Tucker-Lewis index (TLI) (for both: ≥ 0.95 good fit, ≥ 0.90 adequate fit), and the standardized root-mean-square residual (SRMR; < 0.05 good model fit, 0.05 to 0.09 adequate fit) [59, 60]. To achieve better model fit to the data, modification indices were allowed [59].

Convergent validity and divergent validity between Mini-BESTest and other measures (hypotheses 1–4) were assessed using Spearman rank correlation [29]. In addition to the analyses for the total sample, the correlations were assessed for females and males separately. For correlation coefficients, the following criteria were used: > 0.70 indicates convergent validity and < 0.50 indicates divergent validity [30]. Participants missing a PROM were excluded from the analyses, including that specific PROM.

Internal consistency was evaluated for the model with the best fit using Cronbach’s alpha [29]. The items were considered sufficiently correlated if the α coefficient was > 0.70 [61].

## Results

**Fig. 1:**
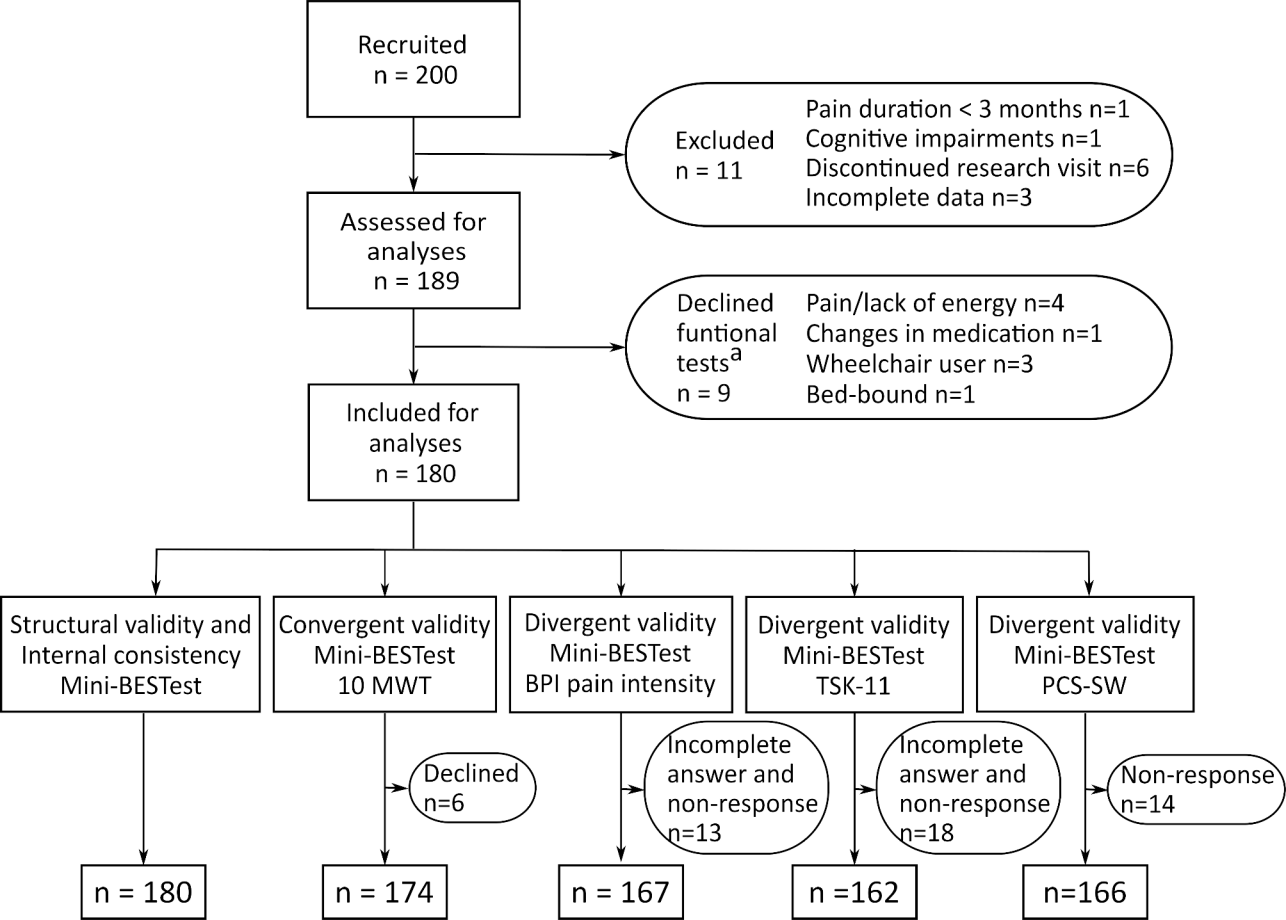
Flowchart of the included and excluded data ^a^ Declined both the Mini-BESTest and the 10 m Walk Test. Mini-BESTest = Mini-Balance Evaluation Systems Test. 10 MWT = 10 m Walk Test, both comfortable and maximum walking speed. BPI = Brief Pain Inventory. TSK-11 = Tampa Scale for Kinesiophobia-11. PCS-SW = Pain Catastrophizing Scale - Swedish version

Reasons for exclusions, respectively, declined functional tests, among the 200 individuals included in the study are presented in Fig. 1. In total, 180 individuals (112 females, 68 males; mean age 51.6, SD 15.9) were assessed with the Mini-BESTest and included in the analyses (Table 1).

**Table 1:** Sociodemographic and clinical characteristics of all participants, separated by sex

	Units	Overall	Female	Male
Sex, n = 180	n (%)	180 (100)	112 (62.2)	68 (37.8)
Age (y), n = 180	mean (SD)	51.6 (15.9)	50.7 (15.5)	53.1 (16.5)
Nation of birth, n = 173	n (%)			
Sweden		150 (86.7)	95 (88.8)	55 (83.3)
Other European country		15 (8.7)	8 (7.5)	7 (10.6)
Non-European country		8 (4.6)	4 (3.7)	4 (6.1)
Education, n = 173	n (%)			
<High school		48 (27.7)	21 (19.6)	27 (40.9)
High school		77 (44.5)	48 (44.9)	29 (43.9)
University or college		48 (27.7)	38 (35.5)	10 (15.2)
Main occupation, n = 169	n (%)			
Employment		49 (27.2)	33 (31.1)	16 (25.4)
Student		2 (1.1)	1 (0.9)	1 (1.6)
Retired ^a^		60 (33.3)	34 (32.1)	26 (41.3)
On long-term sick leave ^b^		44 (24.4)	30 (28.3)	14 (22.2)
Unemployed		7 (3.9)	4 (3.8)	3 (4.8)
Other		7 (3.9)	4 (3.8)	3 (4.8)
Referral to, n = 180	n (%)			
Outpatient pain consultation		121 (67.2)	80 (71.4)	41 (60.3)
Inpatient multimodal pain assessment or rehabilitation		59 (32.8)	32 (28.6)	27 (39.7)
Pain duration, n = 177	n (%)			
3 months – 1 year		6 (3.4)	4 (3.6)	2 (3.0)
> 1 year – 3 years		32 (18.1)	16 (14.5)	16 (23.9)
> 3 years – 10 years		50 (28.2)	32 (29.1)	18 (26.9)
> 10 years		89 (50.3)	58 (52.7)	31 (46.3)
Pain classification ^c^, n = 180	n			
Primary chronic pain		93	65	28
Chronic cancer-related pain		3	0	3
Chronic postsurgical or posttraumatic pain		52	35	17
Chronic neuropathic pain		40	21	19
Chronic secondary headache or orofacial pain		5	4	1
Chronic secondary visceral pain		11	9	2
Chronic secondary musculoskeletal pain		51	38	13
More than one pain condition		63	49	14
Pain intensity ^d^, n = 167	median (Q_1_-Q_3_)	6.5 (5.5–7.2)	6.5 (5.5–7.5)	6.5 (5.5–7.2)
Pain interference ^d^, n = 168	median (Q_1_-Q_3_)	6.8 (5.4–8.3)	6.6 (5.2–8.3)	7.4 (5.4–8.3)
Fear of movement/(re) injury ^e^, n = 162	median (Q_1_-Q_3_)	24.0 (19.0–30.0)	23.0 (17.0–29.0)	26.0 (20.0–32.0)
Pain Catastrophizing ^f^, n = 166	median (Q_1_-Q_3_)	24.5 (15.8–32.0)	24.0 (16.0-33.5)	25.0 (15.0-36.5)
Balance ^g^, n = 180	median (Q_1_-Q_3_) (min-max)	21.0 (16.0–25.0) (1–28)	21.0 (16.0–24.0) (1–27)	22.5 (16.0–25.0) (2–28)
Walking speed (m/s) ^h^, n = 174	mean (SD)			
Comfortable		1.1 (0.3)	1.1 (0.3)	1.1 (0.3)
Maximum		1.5 (0.5)	1.5 (0.5)	1.6 (0.5)

^a^ By age, disability, or early retirement. ^b^ More than 3 months. ^c^ According to the IASP classification of chronic pain for the ICD-11. Based on data from medical records. More than one pain condition per patient is possible. ^d^ BPI-SF = Brief Pain Inventory short form. Pain intensity, composite score including items 3–6, ranges 0–10, with higher scores indicating a higher pain intensity. Pain interference, composite score including item 9a–g, ranges 0–10, with higher scores indicating a higher pain interference. ^e^ TSK-11 = Tampa Scale for Kinesiophobia-11, ranges 11–44, with higher scores indicating a greater fear of movement/(re) injury. ^f^ PCS-SW = Pain Catastrophizing scale – Swedish version, ranges 0–52, with higher scores indicating greater pain catastrophizing. ^g^ Mini-BESTest = Mini-Balance Evaluation Systems Test, ranges 0–28, with higher scores indicating a better balance performance. ^h^ 10MWT = 10 m Walk Test. An average of three trials was used for the calculation of walking speed in meters per second

### Interpretability

As shown in Table 2, the sample was distributed over the whole range of scores of the Mini-BESTest. Twenty-four participants declined to perform one or more items included in the Mini-BESTest. Compared to outpatients, a higher proportion of inpatients declined to perform one or more items (7.4% vs. 25.4%). Of all participants, one participant (0.6%) received the lowest total score, and four participants (2.2%) the highest total score. The total scores were not normally distributed (skewness − 1.0).

**Table 2:** Distribution of scores and number of individuals declining each item

			Distribution of the sample (n = 180)				
			**% over the response options**				
Item	Content of the item	**Declined to perform item** ^a^ n (%) of 180	0	1	2	Mean	SD
	*Anticipatory postural adjustments*						
1	Sit to stand	1 (0.6)	6.1	10.0	83.9	1.8	0.5
2	Rise to toes	3 (1.7)	19.4	40.6	40.0	1.2	0.7
3	Stand on one leg	4 (2.2)	23.9	30.0	46.1	1.2	0.8
	*Reactive postural control*						
4	Compensatory stepping correction - forward	11 (6.1)	14.4	23.3	62.2	1.5	0.7
5	Compensatory stepping correction - backward	15 (8.3)	19.4	36.7	43.9	1.2	0.8
6	Compensatory stepping correction - lateral	20 (11.1)	25.6	38.3	36.1	1.1	0.8
	*Sensory orientation*						
7	Stance (feet together); eyes open, firm surface	2 (1.1)	1.1	7.2	91.7	1.9	0.3
8	Stance (feet together); eyes closed, foam surface	5 (2.8)	9.4	31.7	58.9	1.5	0.7
9	Incline stance – eyes closed	3 (1.7)	4.4	17.2	78.3	1.7	0.5
	*Dynamic gait*						
10	Change in gait speed	6 (3.3)	9.4	22.8	67.8	1.6	0.7
11	Walk with head turns - horizontal	5 (2.8)	12.8	21.1	66.1	1.5	0.7
12	Walk with pivot turns	5 (2.8)	17.2	42.8	40.0	1.2	0.7
13	Step over obstacles	9 (5.0)	18.9	24.4	56.7	1.4	0.8
14	Timed up & go with dual task	5 (2.8)	38.9	51.7	9.4	0.7	0.6
Total ^b^		25 (13.9)					

^a^ Individuals that declined to perform one or more items during testing. ^b^ The number of individuals with at least one missing item

### Construct validity

**Fig. 2:**
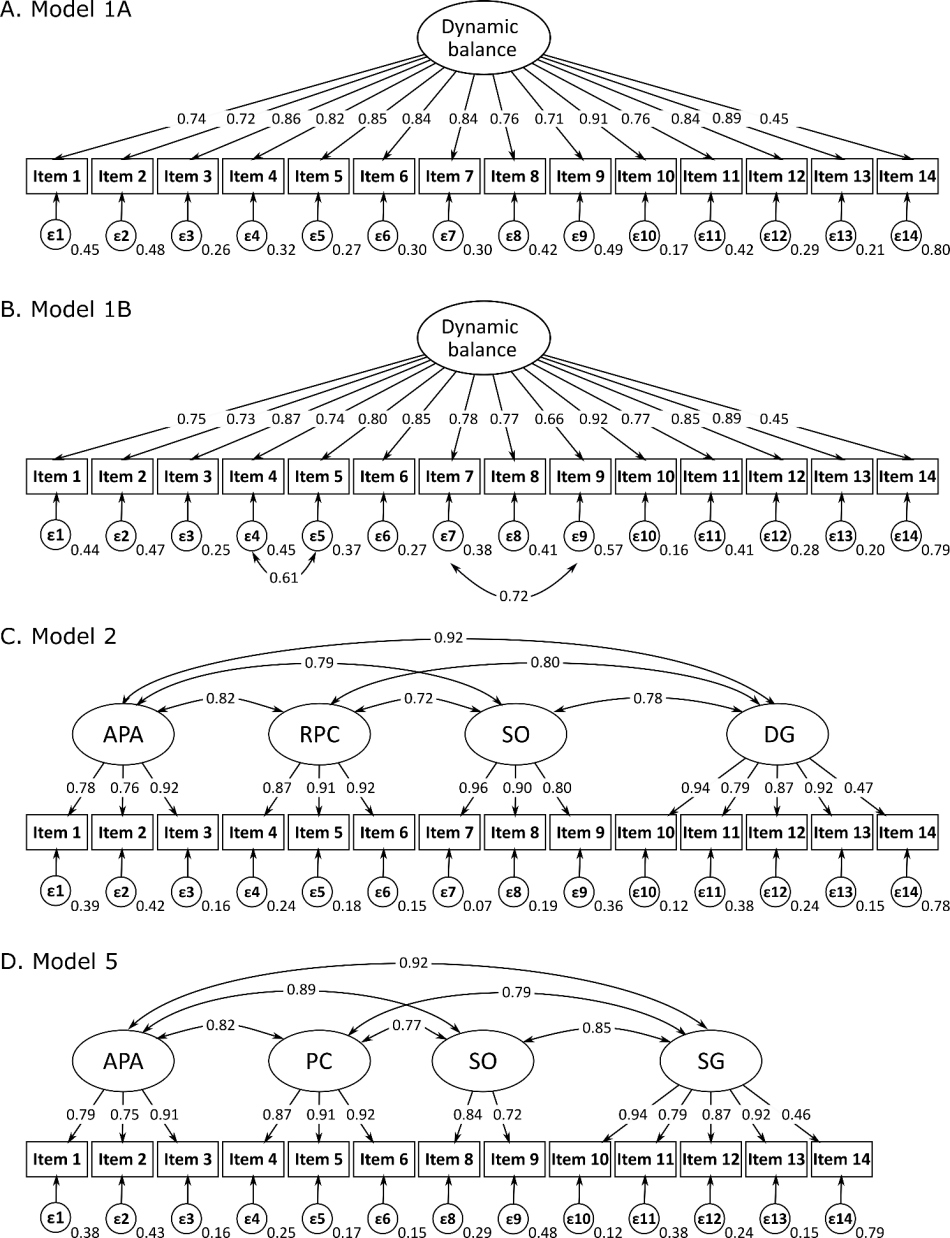
Confirmatory factor analysis for Model 1, Model 2, and Model 5 * Confirmatory factor analysis for the original unidimensional model, presented without added covariance (A; Model 1 A) and with added covariance between items 4–5 and 7–9 (B; Model 1B), a first order 4-factor model based on the four sub-sections in the test (C; Model 2), and a first-order 4-factor model without item 7 (D; Model 5). The factor, i.e., the latent construct, is illustrated by an oval. The item, i.e., the indicator, is illustrated by a rectangle. One-headed arrows from the factors to the items represent the factor loading, and the two-headed arrows represent the covariance between the suggested factors. Each measurement error (ε1–14) is presented by the circle below the item. Item 1 - sit to stand; item 2 - rise to toes; item 3 - stand on 1 leg; items 4, 5, 6 - compensatory stepping correction – forward, backward, lateral; item 7 - stance with feet together and eyes open, on firm surface; item 8 - stance with feet together and eyes closed, on foam surface; item 9 - stance with eyes closed, on an inclined surface; item 10 - walk with change in gait speed; item 11 - walk with horizontal head turns; item 12 - walk with pivot turns; item 13 - step over obstacles; item 14 - timed up & go with dual task. APA = anticipatory postural adjustments; RPC = reactive postural control; SO = sensory orientation; DG = dynamic gait; PR = postural response; SG = stability in gait

In all of the converging models (Models 1, 2, and 5), the factor loadings were > 0.70 for all items, except for item 14 (Fig. 2).

The original unidimensional Model 1 [10] failed the χ ^2^ test, although the other fit indices did meet the thresholds for an acceptable fit (Table 3, Model 1 A). When the covariance was added via the modification indices, the model passed the χ ^2^ test and showed a good overall fit to the data, except for one index that showed an adequate fit (Table 3, Model 1B).

**Table 3:** Goodness-of-Fit indices for confirmatory factor analysis models for Mini-BESTest ^a^

Model	COV	*x* ^2^	*d*ƒ	*p-*value	RMSEA (90% CI)	CFI	TLI	SRMR
1 A:1 factor (14 items)		135.2	77	≤ 0.001	0.07 (0.05–0.08)	0.992	0.993	0.08
1B: 1factor^b^ (14 items)	(4,5); (7,9)	82.8	75	0.25	0.02 (0.00–0.05)	0.999	0.999	0.07
2: 4 factor (APA, RPC, SO, DG)^c^		51.2	71	0.96	0.00 (0.00–0.00)	1.003	1.000	0.06
5: 4 factor (APA, PR, SO, SG)^d^		39.7	59	0.97	0.00 (0.00–0.00)	1.003	1.000	0.05

^a^ Second-order models 3 and 4 did not reach convergence. COV = covariate according to the modification indices between items. RMSEA = root mean square error of approximation; CFI = comparative fit index; TLI = Tucker-Lewis index; SRMR = standardized root-mean-square residual. ^b^ Modification with COV of model 1 A. ^c^ APA = anticipatory postural adjustments; RPC = reactive postural control; SO = sensory orientation; DG = dynamic gait. ^d^ PR = postural response; SG = stability in gait

For Models 2 and 5, high correlations (> 0.90) between the first and the fourth subscale indicates that the different constructs were not measured (Fig. 2). This implies that the models were too complex and therefore overfitting. The interpretation of the models as overfitting was further reinforced by high correlations between the other subscales, as well as fit indices ≤ 0 or > 1 (Table 2, Models 2 and 5) [59]. The two second-order models, Models 3 and 4 [21], did not reach convergence.

Correlations were > 0.70 between Mini-BESTest and 10MWT and < 0.50 between the Mini-BESTest and BPI pain intensity, TSK-11, and PCS-SW, respectively (see Table 4). All correlations were in expected directions and in line with our *a priori* hypotheses for convergent and divergent validity for the total sample, as well as for females and for males.

**Table 4:** Convergent and Divergent validity (Spearman’s rho correlation coefficient) between the Mini-BESTest and other scales

	Mini-BESTest					
	**Total**		**Females**		**Males**	
Scale	r_s_	*p* value	r_s_	*p* value	r_s_	*p* value
10MWT ^a^ – comfortable walking speed	0.72	≤ 0.001	0.71	≤ 0.001	0.73	≤ 0.001
10MWT – maximum walking speed	0.76	≤ 0.001	0.76	≤ 0.001	0.75	≤ 0.001
BPI pain intensity ^b^	-0.22	0.004	-0.13	0.18	-0.35	0.01
TSK-11^c^	-0.06	0.49	-0.11	0.29	-0.04	0.78
PCS-SW ^d^	-0.05	0.52	-0.06	0.57	-0.07	0.60

Mini-BESTest = Mini-Balance Evaluation Systems Test. ^a^10MWT = 10 m Walk Test. ^b^ BPI = Brief Pain Inventory, pain intensity composite score. ^c^ TSK-11 = Tampa Scale for Kinesiophobia-11. ^d^ PCS-SW = Pain Catastrophizing Scale – Swedish version

### Internal consistency

The overall internal consistency of the scale for the unidimensional model 1 was good, according to Cronbach’s alpha (α = 0.92).

## Discussion

This study is the first to evaluate the construct validity and internal consistency of the Mini-BESTest in a chronic pain population in specialized pain care. Our results show that the Mini-BESTest has satisfactory internal consistency and construct validity for the sample. Data supported the finding that the original one-factor model with added covariance via the modification indices had good internal consistency and fit the data from a chronic pain sample well and was also better than the alternative models. In line with our pre-defined hypotheses, Mini-BESTest showed convergent validity with 10MWT, and divergent validity with BPI pain intensity on average, TSK-11, and PCS-SW. Secondly, there were no sex differences in relation to the criteria for convergent or divergent validity.

The one-factor model for Mini-BESTest (model 1) showed a good overall fit to the data from this sample with chronic pain. This result is in line with both the original [10] as well as more recent studies [16–19], supporting the structural validity of the scale in this new population. In agreement with a confirmatory factor analysis performed by Godi et al. [18], adding covariance between specific items, suggested by the modification indices, improved the model fit to the data. In scales with multiple items, some items may share a common variance beyond the variance explained by the latent factor. Thus, adding covariance between error terms for items sharing a common method can be a way to explain redundancy between items [60]. Furthermore, the one-factor model showed high internal reliability (α = 0.92), which is similar to the values reported in other populations (α = 0.89–0.96)[11].

In contrast to previous studies [18, 20], none of the first-order 4-factor models (models 3 and 5) showed an acceptable fit. Both high correlations between subscales, and fit indices, support the interpretation of the model as overfitting [59]. In line with Godi et al. [18], neither of the second-order models (models 3 and 4) reached convergence. To summarize, models with subscales were interpreted as ill-fitting, which implies that the subscales measure the same construct. We, therefore, propose using the total score, instead of the subscale scores, for individuals with chronic pain.

In all models, the factor loadings were high for all items, except for item 14. As in previous research [18, 20], item 14 did not exceed the threshold in any of the converging models. In item 14, the difference in performance is measured between the single-task Time Up and Go (TUG), and the TUG with a cognitive task, i.e., dual-task interference [62]. Even if dual-task interference is closely related to balance, a lower factor loading could be expected, since the two constructs do not overlap fully. Therefore, we analyzed the one-factor model without item 14, resulting in only negligible differences (supplementary Table 1). Considering that there was no improvement the in structural validity, we do not propose excluding item 14, in accordance with previous studies [18].

Moreover, item 14 adds a clinically important aspect since numerous activities in daily living are performed within dual-task conditions [62]. Dual-task interference has been explored in individuals with chronic pain with some discrepancy as to whether an added dual-task will result in an interference in the motor performance [63, 64], possibly leading to an increased risk for falls [65], or a compromised cognitive performance to optimize balance [66]. Furthermore, some studies suggest that the dual-task has a distracting effect, leading to an improvement in balance [67]. In item 14, the same score is given irrespective of which of the two tasks are being interfered with. To be able to fully interpret the dual-task interference and follow change over time, we suggest also noting if the added dual-task results in a decrease in either the walking speed, or the cognitive task, or both. This would further increase the clinical value of the item [68].

The hypothesis for convergent validity was supported by the correlations above 0.7 between the Mini-BESTest and 10MWT. For two measures of the same construct, one could argue that a higher cut-off score would be preferred [30]. Still, based on the hypotheses on the integrated control of posture and gait [14, 15], the instruments were not expected to measure the same constructs, but rather the overlapping constructs [69]. The relation between the Mini-BESTest and 10 MWT has not previously been examined among individuals with chronic pain. However, comparable correlations have been found in other populations [70].

The hypotheses for divergent validity were supported by the weak to negligible correlations between the Mini-BESTest and BPI pain intensity, TSK-11 and PSC-SW, respectively. Pain catastrophizing and fear of movement are associated with pain-related outcomes, such as disability [71] and activity avoidance [72], which may affect balance. In addition, these constructs have been suggested as possible factors that can influence the physical capacity test in individuals with chronic pain [73]. However, the divergent validity implies that in this population, the Mini-BESTest was capturing a construct with little overlap with the construct measured by the BPI, TSK-11, and PCS-SW.

Based on the criteria for our hypotheses, no floor or ceiling effect was found. These results are in line with a review of the psychometric properties of the Mini-BESTest, where the proportion of the sample that reached the top score ranged from 0.9 to 4.3% [11]. However, the scores in our study were not normally distributed, with a majority on the higher end of the scale, indicating a limitation in being able to detect a change in individuals with higher scores on the scale. As in other studies [16], a large proportion of the sample received the highest score on items 1 and 7, respectively. This indicates that these items do not detect the variations in the level of balance sufficiently, which was also confirmed by the low SD of the item scores.

This study has some limitations and strengths worth considering. The Mini-BESTest and the PROMs were not administered at the same time. Since the PROMs were also part of the clinical routine, all individuals were asked to answer them in advance of their first clinical visit, which was in conjunction with the baseline visit. This might add a risk that either the balance, or the factors measured by the PROMs, could change during the time between assessments. However, in the study, the included PROMs are regarded as relatively stable measures of the factors they assess [11, 41, 52, 74–76]. In addition, the health-related variables were not expected to change significantly during the set period of a maximum of six weeks prior to or after the baseline visit. Nevertheless, this could have affected the correlations found for the divergent validity.

The balance assessments were performed by different raters, which could affect the reliability. Mini-BESTest has shown good to excellent intra-rater reliability in other populations [11, 38]. To further improve the reliability in the current study, all raters were trained before the start of the study. However, more studies are needed to evaluate both the inter-rater reliability and the test-retest reliability for the Mini-BESTest in individuals with chronic pain. In addition, to use the Mini-BESTest to measure any change in balance over time in this population, further studies are needed to validate the responsiveness aspects, such as minimal clinically important change.

During testing, some participants declined to perform one or more items. This might also happen in clinical care. Still, strategies to handle this are not described in the Mini-BESTest manual. In this study, the item declined was scored with 0 (*unable or requiring help to perform*). However, we cannot be sure if the scores represent those individuals’ true balance ability or if their refusal to perform is a result of something else. When comparing participants that declined to perform one or more items with the group with a complete test, the group with non-performers had lower scores on all Mini-BESTest items. Furthermore, the comfortable walking speed in the group of non-performers was lower compared to the group that completed the test (supplementary Table 2). Altogether, this could indicate that the group declined to perform items because of balance impairments.

The Mini-BESTest data were collected in conjunction with routine care, hence, in the setting where the instrument will be used. The fact that the study was performed in a clinical setting is a strength for interpretation of clinical use and research [29]. Furthermore, the sample could be considered representative of the target populations of individuals with severe pain problems, referred to a specialized pain center [77]. The results can therefore be generalized to this specific population and setting.

## Conclusion

In conclusion, our findings support that the Mini-BESTest has adequate internal consistency and construct validity for measuring balance in a sample of individuals with chronic pain referred to specialized pain care. The one-factor model showed an adequate fit. In comparison, models with subscales did not reach convergence, or showed high correlations between subscales, implying that the Mini-BESTest is measuring one construct in this sample. We, therefore, propose using the total score, instead of subscale scores, for individuals with chronic pain. This study is the first to evaluate the psychometric properties of Mini-BESTest in a chronic pain population; hence, further studies are necessary to establish reliability for the Mini-BESTest in the population.

## Electronic supplementary material

Below is the link to the electronic supplementary material.

**Supplementary Table 1**. Goodness-of-Fit indices for confirmatory factor analyses for the one-factor model for Mini-BESTest with item 13 excluded. **Supplementary Table 2**. Descriptive statistics for the Mini-BESTest and the 10-meter walk test for the group with a complete Mini-BESTest and the group with non-performers.

## Data Availability

The datasets used and/or analyzed during the current study are available from the corresponding author on reasonable request.

## Abbreviations

Mini-BESTest: Mini Balance Evaluation Systems test
BPI-SF: Brief Pain Inventory – Short form
TSK: Tampa Scale of Kinesiophobia
PCS-SW: Pain Catastrophizing Scale – Swedish version
BESTest: Balance Evaluation Systems test
COSMIN: Consensus-based Standards for the selection of health Measurement Instrument
PROMs: Patient-reported Outcome Measures
IASP: International Association for the Study of Pain
ICD: International Classification of Diseases
10MWT: 10-meter walk test
CWS: Comfortable walking speed
MWS: Maximum walking speed
NRS: Numeric rating scale
RMSEA: Root mean square error of approximation
CFI: Comparative fit index
TLI: Tucker-Lewis index
SRMR: Standardized root-mean-square residuals
APA: Anticipatory postural adjustments
RPC: Reactive postural control
SO: Sensory orientation
DG: Dynamic gait
PR: Postural response
SG: Stability in gait
COV: Covariate
TUG: Timed Up and Go
