# Supplementary material for: Construct validity of the Mini-BESTest in individuals with chronic pain in specialized pain care
Source: BMC Musculoskelet Disord. 2023 May 17;24:391. doi: 10.1186/s12891-023-06504-9 (PMC10189956; doi:10.1186/s12891-023-06504-9)
Supplement: Supplementary file 1 — Supplementary Table 1. Goodness-of-Fit indices for confirmatory factor analyses for the one-factor model for Mini-BESTest with item 13 excluded. Supplementary Table 2. Descriptive statistics for the Mini-BESTest and the 10-meter walk test for the group with a complete Mini-BESTest and the group with non-performers. [file 12891_2023_6504_MOESM1_ESM.docx]

**Supplementary Table 1.** Goodness-of-Fit indices for confirmatory factor analyses for the one-factor model for Mini-BESTest with item 13 excluded.

| **Model** |  | **COV** |  | **χ ^2^** |  | ***d*ƒ** |  | ***p-*value** |  | **RMSEA**  **(90% CI)** |  | **CFI** |  | **TLI** |  | **SRMR** |
| --- | --- | --- | --- | --- | --- | --- | --- | --- | --- | --- | --- | --- | --- | --- | --- | --- |
| 1C: 1 factor (13 items) ^a^ |  |  |  | 120.6 |  | 65 |  | <.001 |  | 0.07 (0.05 - 0.09) |  | 0.992 |  | 0.993 |  | 0.09 |
| 1D: 1 factor (13 items) ^b^ |  | (4,5); (7,9) |  | 69.0 |  | 63 |  | .28 |  | 0.02 (0.00 – 0.05) |  | 0.999 |  | 0.999 |  | 0.07 |

^a^ Model 1C = the one-factor model 1 with item 14 excluded. ^b^ Model 1D = model 1C with covariate (COV) according to the modification indices between items. RMSEA = root mean square error of approximation; CFI = comparative fit index; TLI = Tucker-Lewis index; SRMR = standardized root-mean-square residual. ^b^ Modification with COV of model 1A.

**Supplementary Table 2.** Descriptive statistics for the Mini-BESTest and 10-meter walk test for the group with a complete Mini-BESTest and the group with non-performers.

|  |  | Complete  Mini-BESTest  n=156 | |  | Declined to perform Mini-BESTest item ^a^  n=24 | |  |  |  |  |
| --- | --- | --- | --- | --- | --- | --- | --- | --- | --- | --- |
| Measures | | **Mean (SD)** | **Median (Q_1_-Q_3_)** |  | **Mean (SD)** | **Median**  **(Q_1_-Q_3_)** |  | **t** | **df** | ***p* value** ^b^ |
| Mini-BESTest | |  |  |  |  |  |  |  |  |  |
| 1. Sit to stand | | 1.8 (0.4) | 2 (2-2) |  | 1.2 (0.8) | 1 (0.5-2) |  |  |  | <.001 |
| 2. Rise to toes | | 1.3 (0.7) | 1 (1-2) |  | 0.7 (0.7) | 1 (0-1) |  |  |  | <.001 |
| 3. Stand on one leg | | 1.4 (0.7) | 2 (1-2) |  | 0.2 (0.4) | 0 (0-0) |  |  |  | <.001 |
| 4. Compensatory stepping correction - forward | | 1.6 (0.6) | 2 (1-2) |  | 0.6 (0.8) | 0 (0-1) |  |  |  | <.001 |
| 5. Compensatory stepping correction - backward | | 1.4 (0.7) | 2 (1-2) |  | 0.3 (0.5) | 0 (0.0.5) |  |  |  | <.001 |
| 6. Compensatory stepping correction - lateral | | 1.3 (0.7) | 1 (1-2) |  | 0.0 (0.2) | 0 (0-0) |  |  |  | <.001 |
| 7. Stance (feet together); eyes open, firm surface | | 2.0 (0.2) | 2 (2-2) |  | 1.6 (0.6) | 2 (1-2) |  |  |  | <.001 |
| 8. Stance (feet together); eyes closed, foam surface | | 1.6 (0.5) | 2 (1-2) |  | 0.6 (0.8) | 0 (0-1) |  |  |  | <.001 |
| 9. Incline stance – eyes closed | | 1.8 (0.4) | 2 (2-2) |  | 1.2 (0.8) | 1 (1-2) |  |  |  | <.001 |
| 10. Change in gait speed | | 1.7 (0.5) | 2 (2-2) |  | 0.7 (0.7) | 1 (0-1) |  |  |  | <.001 |
| 11. Walk with head turns - horizontal | | 1.6 (0.6) | 2 (1-2) |  | 0.8 (0.9) | 1 (0-2) |  |  |  | <.001 |
| 12. Walk with pivot turns | | 1.3 (0.7) | 1 (1-2) |  | 0.5 (0.6) | 0 (0-1) |  |  |  | <.001 |
| 13. Step over obstacles | | 1.5 (0.7) | 2 (1-2) |  | 0.4 (0.6) | 0 (0-1) |  |  |  | <.001 |
| 14. Timed up & go with dual task | | 0.8 (0.6) | 1 (0-1) |  | 0.3 (0.5) | 0 (0-1) |  |  |  | .004 |
| 10-meter walk test, comfortable walking speed | | 1.1 (0.3) |  |  | 0.7 (0.3) |  |  | 5.6 | 174 | <.001 |

^a^ Individuals that declined to perform one or more items during testing.

^b^ Differences in Mini-BESTest item scores and comfortable walking speed between the group with a complete Mini-BESTest and the group that declined to perform one or more item, measured by Fisher’s exact test and the independent t-test, respectively.
